# **Effect of work schedule flexibility as a moderator in the relationship between job stress and wellbeing in pharmacy practice**

**DOI:** 10.1038/s41598-025-10523-y

**Published:** 2025-07-06

**Authors:** Erum Rehman, Khalid Abdullah Alotaibi, Shazia Rehman, Mehmood Ahmad

**Affiliations:** 1 Department of Mathematics, Nazarbayev University, Astana, Kazakhstan; 2 College of Education, Department of Psychology, Prince Sattam bin Abdulaziz University, Al-Kharj, 11942 Saudi Arabia; 3 Department of Psychiatry, National Center for Mental Disorders, The Second Xiangya, National Clinical Research Center for Mental Disorders, Hospital of Central South University, Changsha, 410011 Hunan China; 4 National Technology Institute on Mental Disorders, Hunan Key Laboratory of Psychiatry and Mental Health, Mental Health Institute of Central South University, Hunan Technology Institute of Psychiatry, Hunan Medical Center for Mental Health, Changsha, 410011 Hunan China; 5 Department of Pharmacology and Toxicology, University of Veterinary and Animal Sciences Lahore, Lahore, 54000 Pakistan; 6 Department of Pharmacology and Toxicology, Faculty of Veterinary and Animal Sciences, The Islamia University of Bahawalpur, Bahawalpur, Pakistan

**Keywords:** Job stress, Hospital, Well-being, Work schedule flexibility, Pharmacist, Pakistan, Psychology, Health care, Health occupations

## Abstract

Hospital pharmacists frequently face significant job stress, adversely impacting their well-being. Work schedule flexibility is often proposed as a potential buffer against job stress, yet its effectiveness among hospital pharmacists remains understudied. This study investigates the moderating effect of work schedule flexibility on the relationship between job stress and well-being among hospital pharmacists in Punjab, Pakistan. A cross-sectional survey was conducted between September 2023 and January 2024, involving 414 pharmacists working in public hospitals in Punjab province. The study utilized standardized scales to measure well-being, stress levels, and work schedule flexibility among the participants. Bivariate correlation and multiple regression analyses examined the relationships and interaction effects among the study variables. Bivariate correlation analysis revealed a negative association between job stress and well-being, a positive association between work schedule flexibility and well-being, and a negative association between work schedule flexibility and job stress. Multiple regression analysis indicated that work schedule flexibility significantly moderated the relationship between job stress and well-being, with the interaction term showing a significant effect. The findings underscore the importance of work schedule flexibility in mitigating the negative effects of job stress on the well-being of hospital pharmacists. Implementing policies that enhance work schedule flexibility may serve as an effective strategy to improve pharmacists’ well-being and job satisfaction. Further research is needed to explore these dynamics in healthcare settings and among diverse professional groups.

## Introduction

The well-being of healthcare professionals is a cornerstone of effective, ethical, and sustainable patient care. In high-pressure and rapidly evolving healthcare environments, protecting the physical, mental, and emotional health of providers is essential for maintaining professionalism, clinical accuracy, and system efficiency^1^. Among these professionals, hospital pharmacists serve a frontline role by ensuring the safe and appropriate use of medications, advising on therapy, and upholding clinical standards^2^. However, the complexity and critical nature of their responsibilities often expose them to elevated levels of job stress. Job stress arises when the demands of a role exceed an individual’s perceived ability to cope, leading to harmful emotional and physical responses^3^. In the case of hospital pharmacists, such stress has been associated with burnout, reduced job satisfaction, and impaired overall well-being. Well-being in this context refers to a comprehensive state of mental, physical, and emotional health, encompassing elements such as job satisfaction, emotional stability, physical functioning, and work-life balance^4^.

Healthcare settings are typically characterized by chronic time pressure, high workloads, and the need for meticulous attention to detail^5^. For hospital pharmacists, the stakes are particularly high: even minor errors in medication dispensing can result in severe consequences for patient health^6^. The cumulative burden of these responsibilities may lead to exhaustion, professional dissatisfaction, and long-term psychological and physiological harm, including anxiety, depression, and cardiovascular risk^7,8^. Maintaining well-being across all dimensions—emotional, physical, cognitive, and relational—is thus vital not only for practitioners but also for the quality and safety of patient care^6,9–11^. The tenet that healthcare provision is a universal entitlement underscores the imperative to foreground the welfare of those delivering healthcare services, aiming to guarantee uninterrupted and superior care for practitioners and patients alike^12^. Empirical studies have indicated that when healthcare practitioners prioritize their well-being, they exhibit heightened commitment and efficiency in their professional settings, resulting in enhanced patient care outcomes^13–15^.

Growing recognition of this issue has led to calls for organizational interventions aimed at reducing stress among healthcare workers. One such intervention is the provision of job resources, particularly work schedule flexibility, which refers to the degree of control employees have over the timing and structure of their work^16,17^. Research indicates that flexible scheduling can mitigate the negative impact of occupational demands on health, enhance job satisfaction, and improve productivity. However, its effectiveness may vary depending on job role, rank, and organizational context.

The concept of schedule flexibility—whether spatial (location-based) or temporal (time-based)—has gained increasing attention worldwide^18^. In hospital settings, this may involve rotating shifts, part-time schedules, remote consultation options, or self-managed shift planning^19^. From a managerial standpoint, flexibility also aligns with institutional goals, allowing organizations to adapt operations while maintaining workforce performance^20^. Strategies such as contract-based roles, job rotation, and quality improvement teams have emerged as tools for enhancing flexibility and efficiency. Despite these innovations, healthcare professionals often continue to face long working hours, inflexible schedules, and persistent stress—conditions that restrict their ability to balance professional obligations with personal needs^21,22^.

In Pakistan, where the public healthcare system is under-resourced and overburdened, these challenges are especially acute^23^. The scarcity of human resources in hospitals, combined with rigid scheduling and administrative pressures, exacerbates stress and diminishes job satisfaction for pharmacists and other frontline providers^24^. Introducing adaptable work arrangements in such environments may provide a critical means of preserving professional capacity and improving overall well-being^25,26^. Although implementation depends on institutional willingness and structural constraints, promoting employee-centered scheduling practices is increasingly recognized as a viable approach to supporting healthcare workers and sustaining care quality in demanding systems.

## Theoretical background

### Job stress and well-being

The phenomenon of job stress has received widespread attention due to its significant impact as a critical determinant of mental well-being in the working population^27^. Stress manifests when the demand imposed upon an individual in the professional environment surpasses their perceived capacity to effectively manage the circumstances, leading to an adverse physiological or psychological response^28^. Several scholarly works have delineated theoretical frameworks regarding job stress that have significantly influenced the field^29^. For instance, the Job Demands-Resources (JD-R) model offers valuable insight into this relationship. According to the model, job stress arises when job demands surpass an individual’s resources, leading to strain reactions and detrimental outcomes such as decreased well-being^30^. An example of this is a longitudinal investigation conducted by *Tyssen et al. (2000)* in Norway, which revealed that occupational stress constitutes a potential determinant of diminished mental health among employees^31^. Another systematic review encompassing cross-sectional and longitudinal cohort studies identified a robust correlation between job-related stress and adverse mental health outcomes^32^. Hospital pharmacists encounter numerous challenges that demand more efficient work, including the need to review a high volume of prescriptions and the critical and hazardous consequences of medication errors^33–35^. Consequently, their approach needs improvement to meet these demands. Inadequate resources, such as poor working conditions or limited flexibility in scheduling, may increase stress accumulation and result in declining health^36^. Ensuring the well-being of healthcare professionals is vital, incorporating both psychological and physical health. The assessment of mental well-being involves factors such as job satisfaction, motivation, and the identification of mental health issues like anxiety or depression. Ensuring physical well-being is vital for overall health. Prolonged stress can heighten the risk of cardiovascular diseases (CVDs) and weaken the immune system^37^. It is crucial to acknowledge that healthcare professionals, particularly those in frontline roles, function in demanding environments with frequent exposure to emotionally charged situations. These pressures can result in sustained emotional exhaustion, depersonalization, and reduced engagement, ultimately affecting well-being. Their emotional well-being and ability to effectively manage emotions are paramount for providing optimal care. Therefore, the present study hypothesizes:

#### H1

*Job stress is negatively associated with well-being among hospital pharmacists.*

### Work schedule flexibility as a moderator

A growing body of research emphasizes the need to understand the equilibrium between occupational stress and employee well-being^38,39^. One factor increasingly recognized as essential in this balance is work schedule flexibility, which allows employees greater autonomy over when and how they fulfill their job responsibilities^40^. This flexibility may involve discretionary aspects such as selecting work hours or locations, thereby enabling individuals to better balance their professional and personal obligations.

From a theoretical perspective, work schedule flexibility is rooted in both the Job Demands–Resources (JD-R) model^41^ and the Conservation of Resources (COR) theory^42^. In the JD-R framework, flexibility operates as a job resource—a contextual factor that helps mitigate the effects of high job demands by promoting autonomy and recovery opportunities^41^. Meanwhile, COR theory conceptualizes flexibility as a conservational resource that enables individuals to acquire, protect, and restore valuable psychological and physical assets when threatened by stressors^42^. Together, these models suggest that access to scheduling flexibility can help reduce the adverse effects of job stress and preserve well-being.

Empirical evidence supports this theoretical view, showing that flexible scheduling reduces perceived strain by increasing employees’ perceived control and ability to cope with workplace stressors^43,44^. When individuals are empowered to modify their schedules, they report better stress management, improved job satisfaction, and enhanced overall well-being. This relationship may be particularly pronounced among hospital pharmacists, who often face rigid schedules, limited decision-making autonomy, and intense operational workloads, especially in resource-constrained healthcare systems like Pakistan’s public sector. For these professionals, schedule flexibility may represent a critical coping mechanism, helping to weaken the harmful impact of chronic job stress on well-being. Consequently, the present study hypothesizes:

#### H2

*Work schedule flexibility moderates the relationship between job stress and well-being*,* such that the negative effect of job stress on well-being is weaker when work schedule flexibility is high.*

**Fig. 1:**
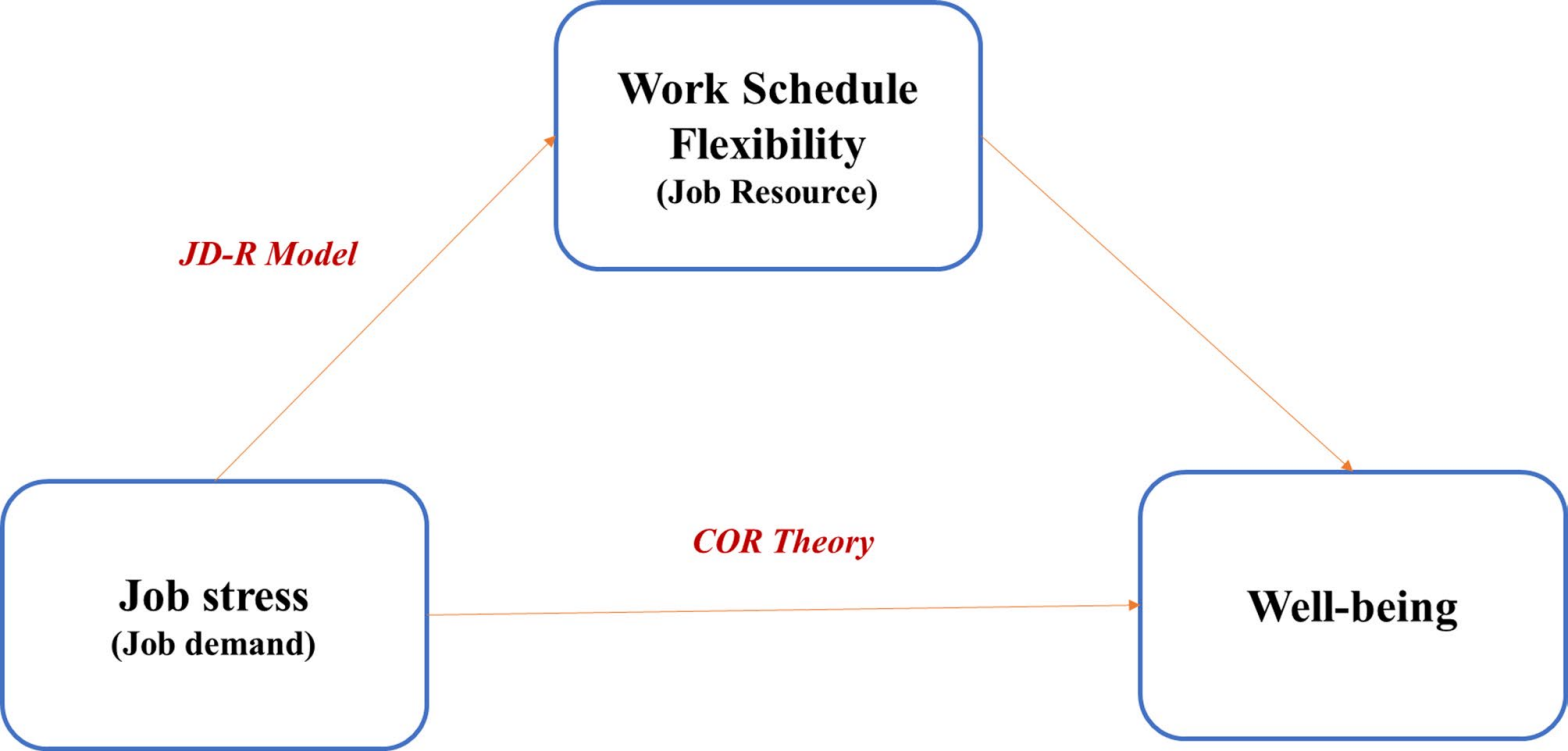
Conceptual model illustrating the moderating role of work schedule flexibility on the relationship between job stress and well-being, grounded in the JD-R and COR frameworks.

### The present study

Despite a substantial body of research on occupational stress and well-being in healthcare^45,46^, there remains a notable gap in the literature specifically addressing the experiences of hospital pharmacists, particularly within low- and middle-income countries (LMICs) such as Pakistan. Much of the existing research has concentrated on physicians and nurses, thereby overlooking the distinct stressors faced by pharmacists, including high prescription volumes, medication-related liability, administrative constraints, and limited decision-making autonomy. These challenges are compounded in Pakistan’s public hospital system, where resource scarcity, rigid scheduling, and workforce shortages further amplify stress and diminish well-being^23,24^. Although job stress has been widely linked to deteriorating mental and emotional health^7,8^, the potential moderating role of job resources, such as work schedule flexibility, has not been thoroughly explored in this professional cohort. Prior studies in other sectors and populations suggest that flexibility in work scheduling can buffer stress and improve well-being^38,44^. However, no empirical research to date has tested this interaction effect among hospital pharmacists in Pakistan or similar LMIC healthcare contexts, a critical oversight in both theory and practice.

Therefore, this study aims to examine the relationship between job stress and well-being among hospital pharmacists working across public hospitals in the Punjab province of Pakistan and to evaluate whether work schedule flexibility moderates this relationship. Specifically, it investigates whether pharmacists with greater schedule flexibility experience weaker negative effects of job stress on their overall well-being. This research addresses a high-priority gap in the literature by focusing on an underrepresented population within an underexplored setting. The study contributes theoretical value by testing an interaction effect grounded in established models of occupational health (JD-R and COR theories) (Fig. 1). It provides practical value by informing hospital policy reforms aimed at reducing burnout, improving work-life balance, and enhancing pharmacist retention. The findings are expected to offer actionable insights for healthcare administrators and policymakers seeking evidence-based strategies to strengthen workforce well-being in resource-constrained environments like Pakistan.

## Methods

### Data source and study participants

A descriptive cross-sectional survey was carried out utilizing stratified sampling across all the public hospitals in Punjab province, Pakistan, between September 2023 and January 2024, wherein each hospital served as a stratum. The study participants (pharmacists) were selected randomly from the registered hospital pharmacist pool furnished by the Pharmacy Council of Punjab, Pakistan. The stratification was intended to achieve a representative sample from a diverse range of hospitals, including those of varying sizes, capacities, and geographic locations in both urban and rural areas of Punjab. The data collection process involved using a structured questionnaire with validated scales, which was sent through online channels such as professional email lists and hospital intranet systems, as well as in person. This approach was employed to facilitate broad accessibility for pharmacists with differing access levels to digital resources.

### Sample size determination

The sample size was determined using Cochrane’s formula to estimate proportions appropriately within a specified margin of error (5%) and confidence interval (95%). The initial sample size was estimated to be 384; however, adjusting for the finite population size of the study population within the selected region, the sample size was reduced to 306. Further, to account for a 25% non-response rate, the final sample size was adjusted to 414, ensuring robustness and representativeness for the study outcomes.

### Eligibility criteria

#### Inclusion criteria

Registered hospital pharmacists actively participating within the Punjab province and willing to participate by completing an informed consent form.

#### Exclusion criteria

Those on extended leave (e.g., maternity, sabbatical) or retired, not currently employed by the hospital, diagnosed with or receiving treatment for psychiatric or psychological disorders, and non-consenting during data collection.

### Data screening and missing values

Before analysis, the dataset was screened for completeness, consistency, and outliers. Responses with more than 10% missing data across critical variables were excluded listwise. For the remaining data, missing values were minimal (< 5%) and handled using mean imputation for scale-based items, a method deemed appropriate given the low proportion and randomness of the missingness. Normality, linearity, and homoscedasticity assumptions were assessed using visual (histograms and Q-Q plots) and statistical tests (e.g., Shapiro-Wilk), confirming suitability for parametric analyses. No multivariate outliers were detected based on Mahalanobis distance thresholds.

### Ethical considerations

The study participants were informed that the data collected from these constructs would remain confidential and only accessible to the researchers and their team. Informed consent has been taken from all the participants. The study has been authorized by the Ethics Review Committee of the University of Veterinary and Animal Sciences, Lahore (No. DR/2043).

### Research instruments

- ***Well-being (Dependent variable)***: The Mental Health Continuum-Short Form (MHC-SF) consists of 14 items that measure emotional (item questions 1–3), psychological (item questions 4–9), and social well-being (item questions 10–14)^47^. Respondents rate each item on how often they have experienced each feeling over the past month on a scale from 0 (Never) to 5 (Every day) (e.g., “During the past month, how often did you feel that your life has a sense of direction or meaning to it”). The MHC-SF has been pre-validated across different populations with excellent internal consistency and test-retest reliability^47–49^.
- ***Job Stress (Independent variable)***: *Cohen (1988)*^50^ developed the Perceived Stress Scale 10 (PSS-10) as a self-reported measure designed to assess individuals’ overall perceived stress levels, delineated into two dimensions: perceived helplessness, reflected in negatively phrased items, and perceived self-efficacy, manifested in positively worded items. Participants were asked to indicate the frequency of their emotions for the previous month using a 5-point Likert scale (*Very often:0* to *Never:4*) (e.g., “In the last month, how often have you been upset because of something that happened unexpectedly?”). To obtain the overall scores, the scoring of positive statements was inverted such that a rating of 4 denoted ‘never’ and a rating of 0 represented ‘very often’. The aggregate score of the PSS-10 spans from 0 to 40, with higher scores indicative of a heightened degree of stress. The PSS-10 scale has been validated in the Urdu version by *Mushtaq*,* Rabia*,* and Riaz*,* Ahmed (2020)*^51^, demonstrating a robust internal consistency coefficient of 0.83. The English version of this measure has been previously validated in the Pakistani community, demonstrating acceptable internal consistency across different demographic groups^52–54^.
- ***Work Schedule Flexibility (Moderator)***: Work schedule flexibility was measured using the Work Design Questionnaire (WDQ) initially developed by *Morgenson and Humphrey (2006)*^55^. To measure the work schedule flexibility of the study participants, we included the flexibility subscale of WDQ, which comprised three items with responses that can be recorded on a 5-point Likert scale from strongly disagrre:1 to strongly agree:5 (e.g., “The job allows me to make my own decisions about how to schedule my work”). The scale has been validated across different populations with excellent internal consistency and test-retest reliability^56–59^.

### Statistical analysis

We initially screened the data on the study variables for normality using the Kolmogorov-Smirnov test to confirm their suitability for subsequent parametric analyses. The constructs’ reliability was evaluated by Cronbach’s alpha for each construct, ensuring internal consistency. Convergent validity was estimated by analyzing each construct’s average variance extracted (AVE), with values > 0.5 considered acceptable^60,61^. The structural model was evaluated using path analysis via structural equation modeling (SEM), facilitating the estimation of associations between job stress, work schedule flexibility, and well-being. The model adequacy was assessed using model fit indices: $\:{\chi\:}^{2}$(df) ratio (< 3 acceptable), Comparative Fit Index (CFI) (acceptable at < 0.95), Tucker-Lewis Index (TLI) (acceptable at < 0.95), and the Root Mean Square Error of Approximation (RMSEA) (acceptable at < 0.08) and Standardized Root Means Squared Residual (SRMR) (acceptable at < 0.06). Pearson bivariate correlation analysis assessed the relationships between the variables in question.

Moreover, a binary logistic regression analysis was employed to assess the correlations between job stress, work schedule flexibility, and the overall well-being of the study participants. Two models were established to investigate the effects among the study constructs. Model 1 represented the crude model, in which no variables were adjusted, while Model 2 was an adjusted model that controlled for age and gender. Then, a multiple linear regression analysis was conducted to examine the associations between job stress and work schedule flexibility with the dependent variable of well-being while controlling for age and gender. The potential moderation effect of work schedule flexibility on the relationship between job stress and well-being was evaluated using regression models incorporating an interaction term (job stress * work schedule flexibility). Prior to regression analysis, the variables of job stress and work schedule flexibility were standardized into z-scores. Statistical analyses were conducted utilizing SPSS version 25, with a significance threshold of *p* < 0.05.

## Results

### Common method bias test

To assess the potential for common method bias (CMB), Harman’s single-factor test was conducted. All measurement items were entered into an unrotated exploratory factor analysis (EFA) to examine the variance explained by a single factor. The results indicated that the first factor accounted for less than 40% of the total variance (i.e., 29.81%), suggesting that common method variance was not a serious concern in this study. This procedure aligns with recommendations from Podsakoff et al. (2003)^62^ for evaluating method bias in behavioral research.

### Reliability and convergent validity analysis results

Table 1 displays the reliability and convergent validity outcomes for the job stress, well-being, and work schedule flexibility constructs. The results of the Cronbach’s alpha analysis revealed high internal consistency for the constructs of job stress (α = 0.85), well-being (α = 0.86), and work schedule flexibility (α = 0.88), indicating robust reliability of the measures used for each of these variables. The composite reliability (CR) values indicate high composite reliability, with job stress exhibiting a value of 0.952, well-being at 0.966, and work schedule flexibility at 0.745. These values imply that the constructs are consistently and dependably measured. The average variance extracted (AVE) values surpass the suggested threshold of 0.50 for all constructs^60,61^, with job stress at 0.666, well-being at 0.677, and work schedule flexibility at 0.693, suggesting strong convergent validity. The findings indicate that the measures employed for these constructs demonstrate reliability and validity, thereby endorsing the utilization of these scales in the research.

**Table 1:** Construct’s reliability and convergent validity results.

	Cronbach’s α	Composite reliability (CR)	Average variance extracted (AVE)
Job stress	0.85	0.952	0.666
Well-being	0.86	0.966	0.677
Work schedule flexibility	0.88	0.745	0.693

### Model fit analysis

Table 2 demonstrates the results of model fit analysis, indicating an acceptable model level for the hypothesized study model. We also evaluated the model fitness for each study construct, and the results showed an acceptable fit to the data. The analysis offers compelling evidence of the adequacy of the measurement and structural model in fitting the data, thereby ensuring the reliability and validity of the constructs under investigation.

**Table 2:** Fit indices of the study model.

Indices	RMSEA	SRMR	CFI	TLI	$\:{\varvec{\chi\:}}^{2}/\varvec{d}\varvec{f}$
Job stress	0.041	0.038	0.951	0.932	1.707
Well-being	0.045	0.040	0.944	0.938	1.87
Work schedule flexibility	0.049	0.042	0.955	0.946	1.93
Overall	0.049	0.041	0.953	0.943	1.99

### Descriptive and confirmatory factor analysis results

Table 3 provides a comprehensive overview of the descriptive statistics and confirmatory factor analysis (CFA) findings about the constructs of Job Stress (JS), Well-being (WB), and Work Schedule Flexibility (WSF). The average score for Job Stress is 32.45, with a standard deviation of 8.91. The distribution shows a slight positive skewness of 0.24 and a moderate kurtosis of -0.45, suggesting that most participants reported higher levels of job stress. The measure of well-being yields a mean score of 22.31 (SD = 6.64), indicative of a minor negative skewness (-0.34) and a more flattened distribution (-0.53), which may suggest reduced levels of well-being. The average score for work schedule flexibility is 28.25 (SD = 4.73), exhibiting a slight negative skewness (-0.49) and nearly normal kurtosis (0.12). These results suggest moderate flexibility in work schedules among the surveyed participants. The results of the CFA indicate robust factor loadings for all items, with values ranging from 0.758 to 0.872 for Job Stress, 0.763 to 0.874 for Well-being, and 0.817 to 0.854 for work schedule flexibility. The substantial factor loadings validate the items as dependable markers of their associated constructs, thus indicating strong convergent validity for the measurement model.

**Table 3:** Descriptive statistics and confirmatory factor analysis (CFA) of the study variables.

Variables with items	Mean	SD	Skewness	kurtosis	Factor loadings
***Job Stress (JS)***	32.45	8.91	0.24	-0.45	
*JS-1*					0.768
*JS-2*					0.826
*JS-3*					0.758
*JS-4*					0.851
*JS-5*					0.812
*JS-6*					0.832
*JS-7*					0.866
*JS-8*					0.793
*JS-9*					0.775
*JS-10*					0.872
***Well-being (WB)***	22.31	6.64	-0.34	-0.53	
*WB-1*					0.778
*WB-2*					0.842
*WB-3*					0.874
*WB-4*					0.781
*WB-5*					0.831
*WB-6*					0.827
*WB-7*					0.763
*WB-8*					0.789
*WB-9*					0.872
*WB-10*					0.844
*WB-11*					0.817
*WB-12*					0.795
*WB-13*					0.838
*WB-14*					0.862
***Work schedule flexibility (WSF)***	28.25	4.73	-0.49	0.12	
*WSF-1*					0.854
*WSF-2*					0.817
*WSF-3*					0.826

### Bivariate correlational analysis results

Table 4 presents the bivariate correlation analysis results, revealing statistically significant associations between job stress, well-being, and work schedule flexibility. The results indicate a significant negative correlation between job stress and well-being (*r* = -0.52, *p* < 0.001) among hospital pharmacists, suggesting that increased job stress is linked to decreased levels of well-being. The observed negative correlation underscores the adverse implications of job stress on individuals’ psychological and emotional well-being within this professional context. Moreover, a negative correlation exists between job stress and work schedule flexibility (*r* = -0.36, *p* < 0.01), indicating that increased job stress is associated with less perceived flexibility in work schedules.

On the other hand, there is a positive correlation between work schedule flexibility and well-being, with a coefficient of 0.45 (*p* < 0.01). The present study demonstrates a positive correlation between increased flexibility in work schedules and elevated levels of individual well-being. The study’s results indicate that increasing the flexibility of work schedules could serve as a practical approach to enhancing the overall well-being of hospital pharmacists. The observed correlations highlight the significance of considering job-related stress and the flexibility of work schedules as pivotal determinants in enhancing the mental health and overall well-being of individuals within this particular professional cohort.

**Table 4:** Bivariate correlational analysis of the study variables.

	Job stress	Well-being	Work schedule flexibility
Job stress	1		
Well-being	-0.52***	1	
Work schedule flexibility	-0.36**	0.45**	1

### Logistic analysis results

The present analysis employs logistic regression to investigate the association between job stress, work schedule flexibility, and overall well-being, as depicted in Table 5. Model 1 demonstrates that heightened levels of job stress are significantly correlated with a higher likelihood of reduced well-being (OR = 1.63, 95% CI: 1.41–2.78), suggesting that elevated job stress levels are linked to a considerable decrease in overall well-being. Moreover, increased flexibility in work schedules has been linked to enhanced well-being (OR = 1.17, 95% CI: 1.02–2.33).

Model 2, after controlling for age and gender, provides additional evidence supporting these findings, demonstrating that job stress continues to be a substantial indicator of decreased well-being (OR = 1.47, 95% CI: 1.29–2.56). Additionally, it illustrates that work schedule flexibility positively impacts well-being (OR = 1.26, 95% CI: 1.05–2.23). The findings indicate that age plays a substantial role, as individuals in the 22–35 age group (OR = 2.51, 95% CI: 1.27–4.36) and 36–45 age group (OR = 2.72, 95% CI: 1.15–4.34) exhibit more significant levels of well-being compared to their counterparts in the 46–60 age group. The findings indicate that the impact of gender on well-being is not statistically significant (OR = 1.39, 95% CI: 0.94–2.37).

**Table 5:** Logistic analysis of job stress and work schedule flexibility with well-being.

		Odds ratio (OR)	95% Confidence Interval
Model 1			
	Job stress (ref: low stress)	1.63	1.41–2.78
	Work schedule flexibility (ref: low flexibility)	1.17	1.02–2.33
***Model 2***			
	Age		
	22–35	2.51	(1.27–4.36)
	36–45	2.72	(1.15–4.34)
	46–60	1.29	(0.61–3.69)
	Female (ref: male)	1.39	0.94–2.37
	Job stress (ref: low stress)	1.47	1.29–2.56
	Work schedule flexibility (ref: low flexibility)	1.26	1.05–2.23

Note: Model 1: Crude model. Model 2: Adjusted for age and gender.

### Multiple linear regression analysis results

Table 6 demonstrates the multiple linear regression analysis outcomes that examined the influence of job stress and work schedule flexibility on overall well-being. According to findings from Model 1, a negative association is observed between job stress and well-being (β = -0.571, *p* < 0.001), suggesting that elevated levels of job stress are linked to reduced levels of well-being. On the contrary, a positive correlation exists between work schedule flexibility and well-being (β = 0.493, *p* < 0.001).

In Model 2, the results demonstrated a negative relationship between job stress and well-being (β = -0.584, *p* < 0.001), as well as a positive association between work schedule flexibility and well-being (β = 0.431, *p* < 0.001). To test the moderating role of work schedule flexibility in the relationship between job stress and well-being, an interaction term was computed by multiplying the standardized values of both variables. The significance and direction of the interaction coefficient were evaluated using hierarchical regression. A significant negative coefficient for the interaction term (β = -0.492, *p* < 0.001) indicated that work schedule flexibility weakens the negative relationship between job stress and well-being, consistent with the hypothesized buffering effect. The statistical model demonstrates a significant explanation of 37% of the variance in well-being, as indicated by an R-squared value of 0.37 and a significant F-statistic of 201.47 (*p* < 0.001), suggesting a robust and reliable fit of the model.

**Table 6:** Multiple linear analysis of job stress and work schedule flexibility with well-being.

	Variables	Well-being		
		β	SE	t
Model 1				
	Job stress	-0.571	0.051	11.196***
	Work schedule flexibility	0.493	0.048	10.271***
	*R* ^*2*^	*0.23*		
	*F*	96.58***		
***Model 2***				
	Job stress	-0.584	0.049	11.92***
	Work schedule flexibility	0.431	0.044	9.80***
	Job stress * Work schedule flexibility	-0.492	0.053	9.28***
	*R* ^*2*^	*0.37*		
	*F*	201.47***		

Note: ***p < 0.001. Both models adjusted for age and gender. The job stress and work schedule flexibility were transformed into z-scores for moderating effect analysis.

## Discussion

This study investigated how flexible work schedules can impact the relationship between job stress and well-being among hospital pharmacists in Pakistan. The study was motivated by the high-stress working conditions that hospital pharmacists experience, which can significantly impact their well-being and job performance. The main goal was to determine whether flexible work schedules could help to reduce the adverse effects of job stress on well-being. The key findings show that higher job stress is linked to lower well-being, while greater work schedule flexibility is associated with improved well-being. Importantly, the research indicates that flexible work schedules can moderate the relationship between job stress and well-being, suggesting that these arrangements could help to alleviate the adverse effects of job stress. These findings highlight the importance of implementing flexible work schedules to support the well-being of pharmacists.

The study findings reveal a strong correlation between job stress and decreased well-being in pharmacists. This supports established research demonstrating the negative impact of high job-related stress on mental and physical health, ultimately leading to a decline in overall well-being^63,64^. The JD-R model emphasizes that job stress arises when job demands outweigh the available resources to address those demands in a balanced way^30^. The demanding and rigorous environment of hospital pharmacy work, characterized by extended work hours, high patient caseloads, and strict accuracy mandates, contributes significantly to the heightened stress levels experienced by pharmacy personnel^65^. In Pakistani hospitals, pharmacists often face elevated stress levels due to a variety of cultural and systemic factors, including limited resources and inadequate support^66,67^. These stressors could profoundly affect the well-being of hospital pharmacists in this particular environment^68^. Moreover, according to the COR theory, stress arises when individuals perceive a potential threat to their resources or experience a loss^68^. Inadequate support and increased job demands in the Pakistani healthcare system may lead to a notable depletion of resources, resulting in reduced well-being among pharmacists.

The study outcomes revealed compelling evidence suggesting that offering work schedule flexibility significantly enhances the well-being of pharmacists. This outcome corroborates existing literature that underscores the favorable impact of flexible work schedules in boosting job satisfaction and alleviating burnout^69–71^. By implementing flexible work schedules, pharmacists can effectively manage their professional and personal responsibilities, reducing stress and improving mental well-being. The theory of the Work-Life Border highlights how flexibility in managing work and personal life boundaries can alleviate conflict and ultimately enhance overall life satisfaction^72^. In Pakistan, professionals frequently contend with mounting responsibilities arising from family commitments and societal expectations. Flexible work schedules offer a crucial solution, providing much-needed relief and enhancing overall well-being^73^. Supported by the Effort-Recovery Model, it is evident that adequate recovery time is vital for sustaining well-being and averting burnout^74^.

Work schedules must be flexible to minimize the negative effects of job stress on well-being^75^, suggesting that modifying work schedules could help reduce stress-related health problems. This notion is further supported by the Person-Environment Fit Theory^76^, where workplace environmental supports can enhance the well-being of the workers when their needs correspond to a good fit towards the environment. Given the reality of limited autonomy, rigid work schedules, and hierarchical organizational structures in Pakistan, healthcare administrators should provide hospital pharmacists with flexible work arrangements. Such a change might also relieve work-related stress, directly or indirectly reducing pharmacists’ health imbalances. In line with the Stress Buffering Hypothesis, seeking exterior help for inner distress will protect against other depletions in your life^77^. Therefore, implementing flexible work schedules can be seen as an external resource that supports managing job-related stress.

In conclusion, this study highlights the significance of flexible work schedules in enhancing the overall well-being of hospital pharmacists in Pakistan. Adaptable work schedules play a crucial role in augmenting pharmacists’ psychological well-being and professional fulfillment by diminishing stress levels and fostering a more harmonious balance between work and personal life. The findings imply that hospital administrators must emphasize the institution of flexible scheduling practices to bolster employee welfare, which could result in improved patient care quality. Additional studies are warranted to explore the enduring impacts of such flexible working patterns and to ascertain the optimal strategies for integrating flexibility into diverse healthcare contexts.

### Implications

This study offers meaningful contributions to both theoretical understanding and practical application within the field of occupational health. Theoretically, this study contributes to occupational health theory by extending the JD-R model and COR theory into a new professional and geographic context. The findings support the JD-R model’s buffer hypothesis by showing that work schedule flexibility moderates the negative impact of job stress on well-being, a relationship previously underexplored in hospital pharmacists. Additionally, the results refine COR theory by demonstrating that even in resource-constrained settings, access to low-cost psychological resources like flexibility can help individuals preserve their well-being under stress. By confirming these effects within Pakistan’s public healthcare system, the study highlights the contextual relevance and generalizability of both models, underscoring the importance of incorporating flexible job design into theoretical models of employee well-being.

From a practical and clinical standpoint, the results highlight the urgent need for healthcare institutions, particularly in resource-constrained settings, to prioritize schedule flexibility as a key component of workforce management. Hospital administrators should recognize that rigid scheduling structures contribute to heightened stress and diminished psychological well-being among pharmacists. Integrating flexible scheduling policies, such as shift-swapping arrangements, compressed schedules, or pharmacist-led input in duty planning, may significantly improve job satisfaction and emotional resilience. In turn, these improvements are likely to enhance professional performance and reduce errors in medication dispensing. For policymakers, the findings suggest that flexibility should be incorporated into broader health workforce retention strategies, especially where financial incentives are limited. By acknowledging the psychosocial needs of pharmacists and embedding flexibility into institutional policies, healthcare systems can foster a more sustainable and efficient working environment that ultimately benefits both practitioners and patients.

### Limitations and future research directions

The present study offers significant findings on the moderating impact of work schedule flexibility on the relationship between job stress and well-being among hospital pharmacists in Pakistan. However, it is important to acknowledge the limitations of this study. First, using a cross-sectional design hinders establishing causal relationships among variables. Longitudinal studies are needed to confirm these associations and dig further into the nature of these relationships regarding causality. Second, self-report questionnaires were used to collect the data, which might have created response and social desirability biases. Future research might profit even more from introducing objective job stress and well-being measures. Third, the study was limited in scope to hospital pharmacists from the Punjab region of Pakistan, which may restrict generalizability across different regions or among healthcare professionals within various settings. Fourth, the impact of Pakistani-specific cultural factors on outcomes is important, and other studies exploring these associations are needed across variably culturally diverse settings for result validation. In addition, the research did not control for potential confounding variables (i.e., personal coping strategies, organizational support, and work environment factors) which might have affected the study outcomes. Addressing these variables in the future can more completely uncover how they might be related to job stress and well-being. Also, the male proportion was more than that of females in the sample, which might have led to a gender bias perception or result in the study. Future studies should aim for more of an even split along gender distribution in such efforts so that results could be representative of the larger population. Lastly, although work schedule flexibility was the main moderator investigated in this study, other moderators should be considered (e.g., job control, social support, professional autonomy). Further research should examine the interplay between factors such as moderating variables, which may be subtle interactions that could better explain complex system dynamics underlying pharmacist well-being.

## Conclusion

In conclusion, this research emphasizes the significant importance of flexible work schedules in mitigating the negative impacts of occupational stress on pharmacists’ physical and mental health in Pakistan’s hospitals. The results indicate that elevated levels of job-related stress are strongly correlated with a decrease in overall well-being. However, the implementation of flexible work schedules has the potential to alleviate this adverse effect. This indicates that implementing policies and interventions targeted at improving flexibility in work schedules could be advantageous in fostering the welfare of pharmacists.

From a theoretical perspective, the findings extend the JD-R and Conservation of Resources (COR) models by empirically demonstrating that schedule flexibility functions as a key job resource that buffers stress and promotes well-being. This supports the resource-based view that organizational provisions can enhance resilience to work demands. The research contributes to the growing body of literature highlighting the need for work-life balance within healthcare environments. The study underscores the importance of healthcare organizations prioritizing flexible scheduling options to enhance job satisfaction and overall well-being. Additional investigation is warranted to examine these associations in diverse contexts and across professional groups to extend the generalizability of these findings. Ultimately, these insights offer meaningful guidance for policy-level reforms aimed at retaining a healthy and effective pharmacist workforce in resource-constrained health systems.

## Data Availability

The raw data supporting this study’s findings are available upon reasonable request from the author, Mehmood Ahmad (mehmood.ahmad@iub.edu.pk).
